# Facilitators and Barriers to COVID-19 Vaccination in Vietnamese Americans in Texas: A Survey

**DOI:** 10.21203/rs.3.rs-6762383/v1

**Published:** 2025-06-18

**Authors:** Diana Omenge, Zeeshan Ali, Paul G. Yeh, Angelica Nguyen, Jannette Diep, Shielene Vargas, Saba Siddiqi, Celine Nguyen, Carlos Fuentes, Bich-May Nguyen

**Affiliations:** University of Houston Tilman J. Fertitta Family College of Medicine; University of Houston Tilman J. Fertitta Family College of Medicine; University of Houston Tilman J. Fertitta Family College of Medicine; Boat People SOS Houston; Boat People SOS Houston; University of Houston Tilman J. Fertitta Family College of Medicine; University of Houston Tilman J. Fertitta Family College of Medicine; UT Southwestern Medical School; University of Houston Tilman J. Fertitta Family College of Medicine; University of Houston Tilman J. Fertitta Family College of Medicine

**Keywords:** COVID-19 vaccines, trust, Vietnamese Americans, Asian Americans, vaccination, peers, clean water access

## Abstract

**Background:**

The coronavirus disease 2019 (COVID-19) pandemic has disproportionately affected Asian American communities, highlighting the need to understand the factors that influence vaccination rates, especially within subpopulations. Many trust studies have found that healthcare institutions, peers, and nonmedical health drivers play key roles in shaping vaccination decisions within specific subgroups, underscoring the need to examine these factors among subpopulations like Vietnamese Americans to develop targeted interventions. Unfortunately, Vietnamese Americans, a significant population in Texas, have limited disaggregated data available, a knowledge gap this study seeks to fill.

**Methods:**

The National Institutes of Health (NIH) Community Engagement Alliance (CEAL) Common Survey 2 instrument was used online and via paper in English and Vietnamese. Trained volunteers, outreach events, and local Texas clinics recruited adults of Vietnamese heritage from December 2022 to April 2023. The data were analyzed through multivariable logistic regression.

**Results:**

Of the 425 participants who responded to a survey, the responses of 278 who completed all pertinent questions were included in the analysis. Respondents demonstrated high trust in healthcare providers (AOR [adjusted odds ratio] 2.97, 95% CI: 1.28–6.86; p = 0.011) and in the federal government (AOR 3.02, 95% CI: 1.32–6.88; p = 0.009) for COVID-19 information were associated with increased odds of COVID-19 vaccination. In contrast, high trust in peers at work or school for COVID-19 information (AOR 0.51, 95% CI: 0.22–0.89; p = 0.041) and a pandemic-related challenge of having clean water to drink in the past month (AOR 0.30, 95% CI: 0.13–0.71; p = 0.006) were associated with decreased odds of COVID-19 vaccination.

**Conclusions:**

Trust in healthcare providers and the federal government was associated with increased COVID-19 vaccine receipt among Vietnamese Americans, whereas trust in peers and endorsing COVID-19 challenges decreased COVID-19 vaccine receipt. Understanding the facilitators and barriers to vaccination among Vietnamese Americans can improve COVID-19 health equity and outcomes.

## Background

The coronavirus disease 2019 (COVID-19) pandemic has affected many communities worldwide. In the United States, studies have shown that COVID-19 has significantly impacted people of color [1, 2]. In particular, the Asian American Native Hawaiian Pacific Islander (AANHPI) community is suspected to have experienced exacerbated impacts from the COVID-19 pandemic due to language barriers and limited data collection [2, 3]. Furthermore, data suggest that in 2020, one in seven deaths was associated with COVID-19 in the Asian American community [4]. Moreover, the Vietnamese American population has been shown to have one of the highest COVID-19 incidence rates [5]. Because the AANHPI population is heterogeneous, a great need exists for disaggregated data to evaluate its impacts accurately [6].

COVID-19 vaccination has proven highly effective in preventing infections, hospitalizations, and deaths caused by the virus. Specifically, the COVID-19 vaccine has demonstrated 86.1% effectiveness against infection in individuals aged ≥ 16 years and 95.3% effectiveness in elderly individuals [7]. Vaccination reduces the risk of adverse health outcomes and helps individuals save on medical expenses, such as doctor visits [7, 8]. Surveys conducted before the widespread availability of COVID-19 vaccines revealed that Asian Americans were more likely to intend to get vaccinated compared to other racial/ethnic groups. [9, 10]. However, recent data show that while COVID-19 booster rates are the highest among Asian Americans, the data fail to disaggregate these rates within specific subgroups [11]. Although disaggregated data exist for hepatitis B serologic testing and vaccination, influenza vaccination rates, and pneumococcal immunizations, limited disaggregated data are available for COVID-19 vaccination among Vietnamese Americans [12]. Past studies on factors influencing hepatitis B vaccination in this population identified barriers such as a lack of knowledge, misinformation, and challenges associated with immigration, all of which may similarly impact COVID-19 vaccination rates [13, 14].

While previous studies have investigated factors affecting AANHPI’s willingness to receive COVID-19 vaccination [9], this population’s unique linguistic and cultural diversity necessitates further exploration. The COMPASS study highlighted that a significant portion of Asian Americans and Pacific Islanders (AAPI), including Vietnamese Americans, have limited English proficiency, with 20% reporting difficulty in speaking English and 18.8% in reading [9]. Language barriers can limit access to credible vaccine-related information, contributing to disparities in vaccine uptake. Hence, identifying the facilitators and barriers to COVID-19 vaccination can help increase the effectiveness of preventative strategies in the Vietnamese American population.

Similarly, understanding the factors that have facilitated or prevented the Vietnamese American population from seeking medical care is crucial to improving their health outcomes. Although AANHPI accounts for a significant portion of the population in Texas, data exploring the impact of COVID-19 on Asian Americans, particularly the Vietnamese American population, are scarce [4]. A previous study exploring the factors influencing the willingness of Vietnamese Americans to participate in COVID-19 research trials revealed that this population generally placed greater trust in university hospitals and pharmaceutical companies than in other information sources like physicians or family, friends, and community leaders and was likely to follow their guidelines [15]. Moreover, the same study revealed that people who place greater trust in federal and government bodies are likely to participate in clinical trials investigating COVID-19 treatment among the Vietnamese population [15]. Social media was among the other facilitators motivating the Asian American population to get vaccinated [16], while [17], explicitly studying the Vietnamese American population, mentioned that medical professionals, the government, and faith-based organizations increased the willingness of Vietnamese Americans to receive the COVID-19 vaccination.

This study aims to identify factors that might have encouraged or discouraged the Vietnamese American population in Texas from being vaccinated against COVID-19. We hope to gain insights into the cultural, social, and economic factors influencing COVID-19 vaccination decisions. These findings will be crucial in designing tailored interventions to improve this population’s COVID-19 vaccination rates and overall health outcomes.

## Methods

### Definition of Facilitators and Barriers

This study defined facilitators as factors positively influencing participants’ willingness to receive the COVID-19 vaccine. These included motivations such as the desire to protect family and community, recommendations from healthcare providers, and a perceived sense of safety. Barriers were defined as factors that negatively impact vaccine uptake. These included challenges such as mistrust in healthcare systems, misinformation, lack of vaccine access, and concerns regarding vaccine safety. Both facilitators and barriers were identified through survey responses, and the analysis aimed to explore how these factors, along with sociodemographic characteristics and trust levels, were associated with the actual receipt of the COVID-19 vaccine.

### Survey instruments

Developed by the National Institutes of Health (NIH) Community Engagement Alliance (CEAL), the Common Survey 2 instrument, translation, and back translation process have been described previously [15]. This comprehensive survey comprises 23 questions covering various areas, including social determinants of health, trust in healthcare systems, risk perceptions related to COVID-19, attitudes toward vaccination, testing behavior, disease control measures, and demographic information. Participants were asked, “How much do you trust each of these sources to provide correct information about COVID-19?” to assess the level of trust in various informational sources regarding COVID-19 vaccines. Sources included healthcare providers, coworkers, faith leaders, social and news media, government entities (federal, state, local), Centers for Disease Control and Prevention (CDC), and community organizations. The trust level response categories included “Not at all,” “A little,” “A great deal,” “Don’t know,” “Does not apply,” and “Prefer not to answer,” offering ordinal data on participants’ perceptions of information reliability. The participants’ motivation to receive the vaccine was also assessed. Self-reported COVID-19 challenges included endorsing various social determinants of health categories, such as access to mental health, housing, food, water, medication, transportation, or family care, as being a minor or major challenge at any point during the pandemic. Finally, sociodemographic variables included language preference (Vietnamese or English), age (as a continuous variable per year of increase), insurance status (yes/no), sex (female-yes/no), educational category (up to high school, some college, or college degree or higher), and history of COVID-19 infection (yes/no).

### Inclusion criteria, recruitment, and data entry

The inclusion criteria were self-identified Vietnamese Americans aged 18 years and above residing in Texas who could read and write in English or Vietnamese. Participants were recruited from December 26, 2022, to April 30, 2023. Online recruitment was performed through email, listservs, social media platforms, and an online advertisement from January 18, 2023, to April 30, 2023. Researchers and community-based organizations (CBO) trained eleven volunteers in January and February 2023 to field the survey and collect paper responses. These volunteers and community partners recruited individuals at community health fairs, local clinics, and literacy classes serving the Vietnamese American community. The participants who completed the survey could enter a drawing for one of sixteen $50 Visa gift cards. All paper survey responses were entered in duplicate by research team members S.S. and S.V. and reviewed for quality assurance. Ambiguous responses were reviewed by S.S., S.V., and B.N. and addressed by consensus.

### Statistical analyses

The independent variables in this study included sociodemographic factors such as language preference, age, insurance status, sex, education level, and history of COVID-19 infection. Additionally, COVID-19-related challenges, trust in various informational sources, and motivations for receiving the COVID-19 vaccine were considered independent variables. The dependent variable was the actual receipt of the COVID-19 vaccination.

An unadjusted, univariable logistic regression analysis was used to assess the association of sociodemographic variables, levels of trust (a great deal vs. less than a great deal of trust) in various sources of information, and COVID-19-related challenges in the past month (vs. not endorsing that challenge) on COVID-19 vaccination receipt in this Vietnamese American population. We also performed a multivariable logistic regression analysis that controlled for sociodemographic factors (age, sex, educational attainment, predominate home language, insurance status, and history of COVID-19 infection) to assess for significant associations with the Wald chi-square statistic of our variables on the odds of COVID-19 vaccination receipt in this Vietnamese American population. Stata Version 17 was used to conduct the statistical analyses, with all the statistical significance being based on a 2-sided probability with a 0.05 significance level.

## Results

In total, 420 people participated in the survey. Of these, 278 respondents provided complete responses to questions, which were included in the multivariable logistic regression analysis. Table 1 outlines the demographics, including age, sex, race/ethnicity, socioeconomic status, inclusion in the regression sample, and the rationale for wanting the COVID-19 vaccination.

### Sociodemographic and Trust in COVID-19 Information on Vaccine Receipt Odds

Table 2 shows the logistic regression results with the unadjusted logistic regression analysis on the left side (Model 1) and the adjusted multivariable logistic regression analysis on the right side (Model 2). A great level of trust in the Food and Drug Administration (FDA) for COVID-19 information was associated with significantly increased odds and marginally increased odds of COVID-19 vaccination in the unadjusted model (OR [odds ratio] 2.16, 95% CI: 1.13–4.15; p = 0.021) and adjusted model (AOR [adjusted odds ratio] 2.20, 95% CI: 0.98–4.95; p = 0.057), respectively. A great deal of trust in the CDC (AOR 2.42, 95% CI: 0.99–4.89; p = 0.052) and in community organizations (AOR 2.29, 95% CI: 1.42–3.79; p = 0.095) as a source of COVID-19 information was marginally associated with increased odds of COVID-19 vaccination.

A great level of trust in healthcare providers (AOR 2.97, 95% CI: 1.28–6.86; p = 0.011) and in the federal government (AOR 3.02, 95% CI: 1.32–6.88; p = 0.009) for COVID-19 information was also associated with significantly increased odds of COVID-19 vaccination. In contrast, a great level of trust in COVID-19 information from peers at school or work was significantly associated with decreased odds of being COVID-19 vaccinated (AOR 0.51, 95% CI: 0.22–0.89; p = 0.041).

In the adjusted model, endorsing a COVID-19 challenge of getting clean water to drink versus not having that challenge in the past month was associated with decreased odds of being COVID-19 vaccinated (AOR 0.30, 95% CI: 0.13–0.71; p = 0.006). The challenge of getting enough food to eat (AOR 0.47, 95% CI: 0.20–1.10; p = 0.081) and taking care of my children or other people (AOR 0.46, 95% CI: 0.20–1.03; p = 0.060) were marginally associated with decreased odds of being COVID-19 vaccinated.

**Figure 1:**
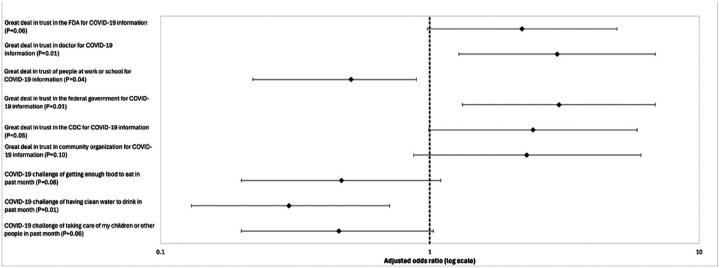
Odds ratios of having at least one dose of the COVID-19 vaccine based on various factors with 95% confidence intervals

Figure 1 provides identified factors that were statistically or marginally statistically significant in this population’s adjusted odds of COVID-19 receipt.

## Discussion

Research on COVID-19 among AANHPIs remains limited, highlighting the need to explore factors influencing vaccine acceptance within this population and specific subgroups, such as Vietnamese Americans. This study addresses this gap by investigating how levels of trust in diverse COVID-19 information sources can influence vaccine willingness. These findings underscore that the origin of COVID-19 information significantly shapes vaccination decisions, independent of demographic variables and pandemic-related barriers. Additionally, the analysis found an association between water insecurity and lower odds of COVID-19 vaccination, suggesting that challenges related to access to basic resources may influence vaccine uptake.

In this context, the study revealed that increased trust in healthcare providers, particularly among participants who relied on primary care providers for COVID-19 vaccination information, was strongly associated with increased vaccine uptake. Vietnamese Americans with high levels of trust in healthcare providers for COVID-19 information had three times the odds of receiving the COVID-19 vaccine than those with lower levels of trust. Previous studies have indicated that having a primary care provider can be beneficial in encouraging patients to seek vaccination [18, 19]. In another study, Peña et al. reported that not having a primary care provider was linked to decreased odds of COVID-19 vaccine uptake in racial and ethnic minority groups [20]. Our finding aligns with those of previous studies, highlighting the critical role that trust in healthcare providers plays in vaccine uptake. Hence, creating policies that can increase access to primary care providers could increase COVID-19 vaccine uptake in Vietnamese Americans.

Furthermore, this study identified a positive association between trust in information from federal agencies, such as the FDA and CDC, and willingness to participate in COVID-19 vaccination, highlighting the possible role of governmental trust in shaping public health engagement. Bacong et al. [16] reported that Asian Americans who relied on official government health websites were significantly less likely to exhibit vaccine hesitancy (OR = 0.46, 95% CI = 0.33–0.62, p < 0.001), suggesting a potential link between government-endorsed information and vaccine confidence. Similarly, Nguyen et al. [17] found that trust in science-based information, particularly when disseminated through culturally competent and community-trusted sources, was associated with greater vaccine acceptance among Vietnamese Americans. Our findings are consistent with prior research indicating that trust in federal government sources is correlated with COVID-19 vaccine uptake. These studies collectively suggest that trust in government information plays a vital role in vaccination behavior, emphasizing the need for transparent and consistent communication to sustain public confidence in future health initiatives.

Unsurprisingly, this study’s result indicated that reliance on peers for COVID-19 information was negatively related to the willingness of the study participants to be vaccinated. Individuals who depended on their peers for vaccine-related information were less likely to get vaccinated. These results could be a product of several factors, such as misinformation, the political affiliation of peers, and the personal beliefs and biases of peers. A study performed by Yu et al. [21] demonstrated that participants’ moral decision-making was influenced by their peers to resemble the preferences of their peers. Similarly, researchers who evaluated the association of race/ethnicity with COVID-19 vaccine uptake reported that people who reported that fewer than all of their peers were vaccinated were less likely to be vaccinated themselves [18]. Several studies have demonstrated that misinformation, political affiliation, and personal beliefs can negatively impact the willingness to be vaccinated and increase vaccine hesitancy among the public [18, 21–23]. Our results are consistent with these studies, suggesting that peer influence may play a significant role in vaccine hesitancy. Understanding the negative impact of peer reliance on vaccine willingness is critical for designing public health interventions. Targeted strategies that address misinformation and leverage trusted, evidence-based sources of information may help mitigate the influence of peers and improve vaccine acceptance.

Our study also found a significant association between water insecurity and decreased COVID-19 vaccination rates. Specifically, individuals who reported difficulty accessing clean drinking water in the past month had 70% lower odds of being vaccinated than those without this challenge. While this association suggests a potential link between water insecurity and vaccination behavior, it does not establish a direct causal relationship. Instead, various social, economic, and structural factors related to water insecurity may contribute to disparities in vaccine uptake. For example, a prior study identified clean water access as a critical challenge indirectly associated with COVID-19 vaccination uptake [24]. Limited access to clean water made it difficult for individuals to practice proper hygiene, such as handwashing, which may have influenced perceptions of COVID-19 prevention [24]. Some individuals, lacking the means to follow hygiene guidelines, questioned the necessity of vaccination or expressed doubts about its effectiveness [24]. Our findings contribute to this body of literature by reinforcing the idea that water insecurity may shape health behaviors beyond hygiene practices. The observed association suggests that individuals facing resource scarcity may experience compounded barriers to preventive healthcare, including vaccination. These results underscore the need for further research to explore the mechanisms underlying this association and to inform targeted public health interventions to reduce vaccine disparities in vulnerable populations.

### Limitations of the study

Several limitations warrant acknowledgment. First, the study design establishes correlations rather than causation. The absence of time frame inquiries in the survey restricts the ability to draw causal conclusions regarding the relationship between trust in sources and vaccine uptake. Additionally, the survey measures may have constrained participants’ ability to express nuanced opinions due to using a narrow Likert scale. Furthermore, the study’s demographics may limit generalizability due to resource constraints, with a low representation of unvaccinated individuals. Lastly, the results also depend on self-reported data, which may be subject to recall bias or misclassification. However, a key strength of the study is its unique contribution to understanding the factors that facilitate and hinder COVID-19 vaccination uptake among Vietnamese Americans, and it can serve as a baseline for similar research within this population.

## Conclusion

This study highlighted the complex interplay of factors influencing COVID-19 vaccine acceptance among Vietnamese Americans. Trust in healthcare providers and the federal government for COVID-19 information was associated with increased odds of vaccine attainment, whereas reliance on peers for COVID-19 information and endorsing the COVID-19 challenge of getting clean water to drink were associated with decreased odds of COVID-19 vaccination. These findings underscore the need for tailored health communication strategies that consider various information sources, build trust in healthcare providers, and promote community-level initiatives and non-medical drivers of health to encourage vaccine acceptance among Vietnamese Americans. Further studies are needed to understand better the underlying mechanisms influencing peer influence. Public health interventions can be more effectively designed to mitigate vaccine hesitancy and improve health outcomes within this population by addressing these areas.

## Figures and Tables

**Table 1:** Participant demographics

Variable	Total sample (n = 425)	Regression sample (n = 278)	p-value*
Age, mean (SD)	51.5 (18.7)	52.3 (18.7)	0.282
Biological female, n (% of total)	203 (56.9)	157 (56.5)	0.931
Educational attainment, n (% of total)			
Up to high school	178 (53.5)	113 (50.7)	0.375
Some college	57 (17.1)	37 (16.6)	
College degree or higher	98 (29.4)	73 (32.7)	
Home language, n (% of total)			
English	137 (37.9)	106 (38.1)	0.839
Vietnamese	225 (62.2)	172 (61.9)	
With health insurance, n (% of total)	314 (88.5)	253 (91.0)	0.755
History of COVID-19 infection, n (% of total)	133 (38.7)	108 (38.9)	0.884
A great deal of trust in the following sources for COVID-19 information, n (% of total)			
U.S. Food and Drug Administration	171 (47.9)	140 (50.4)	0.331
Doctor	231 (66.6)	191 (70.2)	0.256
People at work or school	78 (23.1)	67 (25.2)	0.327
The news	154 (44.9)	126 (46.8)	0.418
Social media	58 (17.9)	48 (18.8)	0.662
Federal government	202 (58.7)	160 (58.8)	0.940
State or local government	198 (57.6)	157 (57.7)	0.906
Centers for Disease Control and Prevention	207 (60.0)	169 (62.1)	0.369
Community organization	118 (35.9)	94 (36.4)	0.682
Challenges experienced in the past month due to the COVID-19 pandemic			
Mental health care	118 (33.3)	87 (31.6)	0.456
Having a place to live	68 (19.2)	58 (20.9)	0.358
Getting enough food to eat	99 (27.7)	70 (25.2)	0.293
Having clean water to drink	76 (21.3)	55 (19.8)	0.443
Getting medications I need	116 (32.5)	78 (28.1)	0.151
Getting where I need to go	132 (37.1)	97 (35.0)	0.282
Taking care of my children or other people	117 (32.8)	83 (29.9)	0.278
Received at least one dose of the COVID-19 vaccine	310 (86.8)	248 (89.2)	0.563

* The chi-square test for categorical variables and the Wilcoxon rank-sum test for continuous variables were used to test for differences in proportions and means. Some variables had participants who did not respond, so we only calculated the proportion among those who responded to that variable.

**Table 2:** Logistic Regression of Sociodemographic, Trust, and Challenges on COVID-19 Vaccine Receipt in Vietnamese Americans

Odds of having had received at least one dose of COVID-19 vaccine	Model 1 (n = 425)^a^				Model 2 (n = 278)^a^			
	Odds Ratio	p-value	95% Confidence Interval		Adjusted Odds Ratio	p-value	95% Confidence Interval	
			Low	High			Low	High
**Age per year increase**	1.01	0.142	0.99	1.03	1.02	0.248	0.99	1.04
**Sex**								
Biological female	0.79	0.473	0.42	1.49	1.00	0.992	0.44	2.27
Not biological female	Reference				Reference			
**Educational attainment**								
Up to high school	Reference				Reference			
Some college	0.56	0.155	0.25	1.24	0.52	0.208	0.19	1.44
College or higher	1.18	0.688	0.53	2.61	0.99	0.979	0.37	2.64
**Home language**								
Vietnamese	1.17	0.630	0.62	2.18	0.93	0.887	0.33	2.61
English	Reference				Reference			
**Insurance status**								
Current health insurance	1.71	0.212	0.74	3.97	2.28	0.139	0.77	6.80
No health insurance	Reference				Reference			
**History of COVID-19 infection**								
Positive history	0.94	0.843	0.49	1.78	0.73	0.468	0.31	1.72
No history	Reference				Reference			
**A great deal vs. <a great deal of trust in the following sources for COVID-19 information**^b^:								
FDA	**2.16**	**0.021**	**1.13**	**4.15**	2.20	0.057	0.98	4.95
Healthcare provider	**2.90**	**0.001**	**1.53**	**5.48**	**2.97**	**0.011**	**1.28**	**6.86**
People at work or school	0.55	0.083	0.28	1.08	**0.51**	**0.041**	**0.22**	**0.89**
News	**2.21**	**0.023**	**1.12**	**4.38**	1.87	0.152	0.79	4.42
Social media	0.88	0.766	0.38	2.02	1.45	0.527	0.46	4.57
Federal government	1.75	0.081	0.93	3.29	**3.02**	**0.009**	**1.32**	**6.88**
State or local government	1.22	0.539	0.65	2.28	1.75	0.165	0.79	3.86
CDC	1.28	0.424	0.67	2.45	2.42	0.052	0.99	5.89
Community organization	1.29	0.478	0.64	2.58	2.29	0.095	0.87	6.08
**Challenges experienced in the past month due to the COVID-19 pandemic vs. not having the challenge**^b^:								
Mental health care	**0.49**	**0.027**	**0.26**	**0.92**	0.51	0.117	0.22	1.19
Having a place to live	0.74	0.428	0.36	1.55	0.76	0.577	0.30	1.97
Getting enough food to eat	0.57	0.085	0.30	1.08	0.47	0.081	0.20	1.10
Having clean water to drink	**0.47**	**0.024**	**0.24**	**0.90**	**0.30**	**0.006**	**0.13**	**0.71**
Getting medications I need	0.61	0.117	0.32	1.13	0.63	0.272	0.28	1.44
Getting where I need to go	**0.51**	**0.035**	**0.28**	**0.96**	0.61	0.239	0.27	1.39
Taking care of my children or other people	**0.45**	**0.013**	**0.24**	**0.85**	0.46	0.060	0.20	1.03

The bolded results indicate statistically significant Wald chi-square statistic (p < 0.05) results.

a Model 1 shows the unadjusted univariable association of each row’s variable with the odds of COVID-19 receipt. Model 2 is a multivariable logistic regression that adjusts each variable for age, sex, educational attainment, home language, insurance status, and history of COVID-19 infection.

b The reference group for trust was any response less than a great deal of trust in the above sources of information. The reference group for COVID-19 challenge categories were individuals who did not endorse the specific COVID-19 challenge.

## Data Availability

The datasets used and/or analyzed during the current study are available from the corresponding author on reasonable request.

## Abbreviations

AANHPI: Asian American, Native Hawaiian, Pacific Islander
AAPI: Asian American and Pacific Islander
AOR: Adjusted Odds Ratio
CEAL: Community Engagement Alliance
CBO: Community-based organizations
CDC: Centers for Disease Control and Prevention
COVID-19: Coronavirus disease 2019
FDA: Food and Drug Administration
NIH: National Institutes of Health
OR: Odds Ratio
